# Synthesis and Characterization of CaFe_2_O_4_: Catalytic and Bactericidal Evaluation at High Temperatures

**DOI:** 10.3390/ma19071458

**Published:** 2026-04-05

**Authors:** Daniel Eduardo Bernal Lozano, Miguel Andrés Perdomo Gutiérrez, Ailton José Moreira, Vinicius Marques Ferreira, João Otávio Donizette Malafatti, Elaine Cristina Paris, Miryam Rincón Joya

**Affiliations:** 1 Departamento de Física, Universidad Nacional de Colombia, Carrera 30 Calle 45-03, Bogotá 11001, DC, Colombia; debernall@unal.edu.co (D.E.B.L.); mperdomog@unal.edu.co (M.A.P.G.); 2 Institute of Chemistry, São Paulo State University (UNESP), Araraquara 14800-060, SP, Brazil; aijomoquim@gmail.com (A.J.M.); marques.ferreira@unesp.br (V.M.F.); 3 Embrapa Instrumentação, Rua XV de Novembro, 1452, São Carlos 13560-970, SP, Brazil; jmalafatti@hotmail.com (J.O.D.M.); elaine.paris@embrapa.br (E.C.P.)

**Keywords:** CaFe_2_O_4_, photo-assisted removal, pechini method, rietveld refinement, ciprofloxacin degradation, antibacterial assessment

## Abstract

CaFe_2_O_4_ is a p-type ferrite semiconductor of interest for photo-assisted environmental remediation due to its narrow band gap and high chemical stability. In this work, CaFe_2_O_4_ powders were synthesized via the Pechini polymeric precursor method and calcined between 550 and 850 °C to investigate the influence of calcination temperature on structural order and material properties. X-ray diffraction combined with Rietveld refinement revealed the progressive stabilization of the orthorhombic *Pnma* phase, accompanied by relaxation of the FeO_6_ octahedral framework. Raman and FT-IR spectroscopies confirmed a significant increase in vibrational coherence with increasing calcination temperature, quantified by a nearly three-fold increase in the global Raman order parameter and phonon lifetimes. Nitrogen physisorption showed a modest specific surface area and a pore system dominated by interparticle meso–macroporosity, typical of thermally treated ferrites. Removal tests using ciprofloxacin under UV-A irradiation showed limited photo-assisted activity, while agar diffusion assays against *Escherichia coli* and *Staphylococcus aureus* revealed no inhibition halos, indicating the absence of detectable antibacterial activity under the experimental conditions employed. Overall, CaFe_2_O_4_ combines photo-assisted response with good structural stability, highlighting its potential as a chemically stable ceramic material with no detectable antibacterial activity under the tested conditions.

## 1. Introduction

Ferrites have long been recognized for their relevance in materials science owing to their tunable optical properties, chemical stability, and structural robustness [1]. Their ability to tune the band gap through compositional control has enabled a wide range of applications, including batteries [2], sensors [3], energy-related technologies [4], and heterogeneous catalysis [1,5,6,7,8,9]. In this context, the increasing demand for sustainable and cost-effective materials has renewed interest in ferrites composed of earth-abundant elements, making calcium ferrite (CaFe_2_O_4_) a particularly attractive yet still underexplored candidate [6].

CaFe_2_O_4_ is a p-type semiconductor with a narrow band gap (1.9–2.1 eV) [6,10,11], providing an optical response well suited for environmentally driven applications. Structurally, the large ionic radius of Ca^2+^ favors its occupation of high-coordination sites, while Fe^3+^ ions are accommodated in distorted octahedral environments [12]. This cationic arrangement leads to an orthorhombic crystal structure characterized by one-dimensional channels, markedly different from the conventional cubic spinel framework. This distinctive crystallographic architecture confers on CaFe_2_O_4_ unique electronic features. Electronic structure calculations indicate that the upper valence band is mainly derived from O 2p states hybridized with Fe 3d orbitals, favoring oxidative pathways, while the lower conduction band is primarily composed of Fe 3d states, providing moderate reducing capability [13]. Together, these characteristics make CaFe_2_O_4_ thermodynamically suitable for photoinduced oxidation processes.

However, the optimization of CaFe_2_O_4_ for environmental applications faces a critical thermodynamic limitation. While obtaining the stoichiometrically pure orthorhombic phase typically requires high calcination temperatures, this thermal treatment promotes grain growth and sintering, leading to a significant reduction in the active surface area and porosity [6,14,15]. Conversely, lower synthesis temperatures may preserve favorable textural properties but often result in the coexistence of metastable intermediates (e.g., CaCO_3_, α-Fe_2_O_3_) [16] or lattice defects [17]. This creates a trade-off where the optimal balance between crystallographic order and surface availability is not always clear. While the photocatalytic performance of CaFe_2_O_4_ has been explored for the degradation of various pollutants [6,18,19] and heterostructured systems [20,21] have been proposed to alleviate charge carrier recombination, the intrinsic relationship between phase purity, microstructural evolution, and functional activity remains insufficiently understood.

From an environmental perspective, the widespread occurrence of pharmaceuticals in aquatic systems and the rise of antimicrobial resistance represent critical global challenges. Ciprofloxacin (CIP), a second-generation fluoroquinolone antibiotic, is frequently employed as a model contaminant due to its high chemical stability and environmental persistence [22,23,24]. This behavior is largely attributed to the high bond dissociation energy of the C–F linkage, the steric hindrance imposed by the piperazine ring, and its zwitterionic nature [22]. Because of the relatively large molecular size and complex ionization of CIP, its interaction with the catalyst surface plays a decisive role in facilitating its degradation. Therefore, materials capable of providing sufficient active sites for adsorption prior to reaction are desirable. Moreover, the persistence of CIP in aquatic environments and its potential bioaccumulation raise significant concerns regarding ecosystem integrity and public health [23,24]. Consequently, probing the structural and textural evolution of CaFe_2_O_4_—and its impact on surface affinity—provides a useful way to evaluate its functional behavior.

In this context, the polymeric precursor (Pechini) route was selected because it promotes homogeneous mixing of the metal precursors at the molecular level and allows improved compositional control during oxide formation [25]. Furthermore, determining the behavior of this ferrite requires going beyond the analysis of the final crystalline phase to systematically explore its thermal evolution and defect structure. Specifically, investigating these structural features in the context of ciprofloxacin removal is an important step to validate its practical relevance in environmental remediation.

This study explores the structural, vibrational, and optical evolution of CaFe_2_O_4_ synthesized via the Pechini method, with emphasis on the effect of calcination temperature (550–850 °C) on the balance between lattice relaxation, structural disorder, and surface area. A multi-technique characterization approach was employed, including thermal (TG, DTG, DSC), structural and vibrational (XRD, Raman, FT-IR), morphological (FEG-SEM), textural (N_2_ physisorption), and optical (UV-Vis) analyses. Based on this characterization, the suitability of the material for ciprofloxacin removal under UV-A irradiation was evaluated, together with a preliminary investigation of its interaction with model bacterial strains (*E. coli* and *S. aureus*). This approach allows correlating the structural and textural evolution of CaFe_2_O_4_ with its functional behavior in environmental applications.

## 2. Materials and Methods

### 2.1. Synthesis of CaFe_2_O_4_ Using the Pechini Method

CaFe_2_O_4_ was synthesized using the Pechini polymeric precursor method, which enables homogeneous mixing of metal ions at the molecular level through chelation and polyesterification reactions. Calcium acetate (PANREAC, Castellar del Vallès, Spain) and iron(III) nitrate (CHEMÍ, Bogotá, Colombia) were used as metal precursors, while citric acid and ethylene glycol (PANREAC, Castellar del Vallès, Spain) were used as the chelating agent and polymerization medium, respectively.

Initially, 0.79 g of calcium acetate, Ca(CH_3_COO)_2_, was dissolved in 20 mL of distilled water at room temperature. Separately, 4.04 g of iron(III) nitrate nonahydrate, Fe(NO_3_)_3_·9H_2_O, was dissolved in 30 mL of warm distilled water. Both solutions were stirred for 15 min at 510 rpm and 50 °C for complete dissolution. Then, 4.3 g of citric acid monohydrate was dissolved in distilled water and added to the mixed calcium–iron solution under continuous stirring to form metal–citrate complexes.

Ethylene glycol (3.9 mL) was then added to the solution to induce polyesterification between citric acid and ethylene glycol. The resulting solution was stirred for an additional 5 min, and the pH was adjusted to the range 2–4 for stable chelation and polymer network formation. The homogeneous sol was subsequently dried at 120 °C for 9 h, followed by an additional drying step of 12 h at the same temperature, yielding a polymeric resin precursor.

The dried precursor was calcined in air at different temperatures (550, 650, 750, and 850 °C) for 3 h to obtain crystalline CaFe_2_O_4_ and to investigate the influence of thermal treatment on phase formation and functional behavior. Phase purity and crystallinity were confirmed by X-ray diffraction (XRD). Based on the structural and surface characteristics, selected samples were further evaluated for bactericidal activity and photocatalytic performance. The reaction equations were balanced using the open-access platform WebQC (https://es.webqc.org/balance.php, accessed on 15 September 2025).

### 2.2. Ciprofloxacin Removal and Photo-Assisted Reactivity

The removal performance of the materials was evaluated in 100 mL of a 1 mg L^−1^ ciprofloxacin solution (CIP, Sigma-Aldrich, St. Louis, MO, USA, ≥98%) with a catalyst concentration of 0.5 g L^−1^. Initially, the mixture was kept in the dark for 30 min to reach adsorption–desorption equilibrium, and then the UV-A (365 nm) light source was turned on. This dark stage allows the contribution of physical adsorption to be evaluated prior to irradiation. For the kinetic study, the selected materials were tested under the same conditions, with degradation times ranging from 0 to 180 min. The dimensions of the photocatalytic reactor are described in Da Silva Ribeiro et al. [26].

### 2.3. Halo of Inhibition—Qualitative Antibacterial Assay

The antibacterial activity of the synthesized materials was evaluated using a qualitative inhibition halo assay based on an agar surface diffusion method. Initially, bacterial strains were cultivated in a culture medium and incubated at 35 °C for 24 h under shaking to ensure homogeneous bacterial growth. After incubation, the bacterial concentration was standardized by UV-Vis spectrophotometry at 650 nm. Turbidity was adjusted according to the McFarland standard, corresponding to approximately $1.6\times 10^{7}$ CFU mL^−1^. Subsequently, the inoculum was diluted in fresh nutrient medium, and 100 μL of the standardized suspension was uniformly spread onto the surface of nutrient agar plates. After inoculation, 5 mg of each material was placed at a single point on the agar surface.

**Figure 1:**
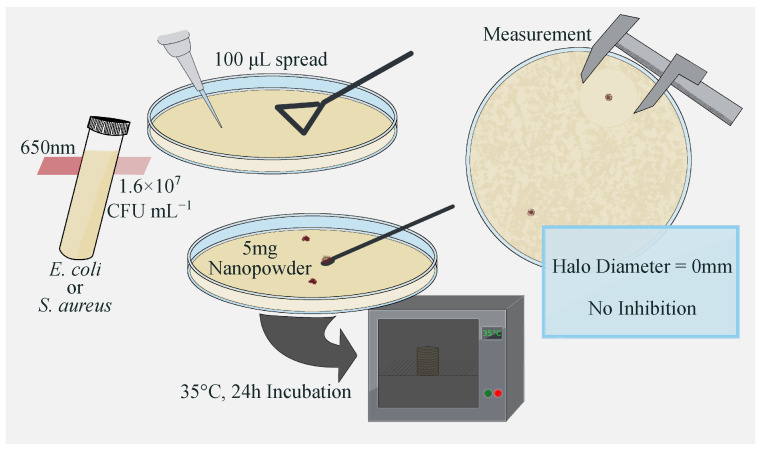
Schematic illustration of the agar diffusion method used to evaluate antibacterial activity.

The inoculated plates were incubated at 35 °C for 24 h. After incubation, the formation of a clear inhibition halo around the material indicated the suppression of bacterial growth. The antibacterial effect was qualitatively evaluated by measuring the halo diameter (Figure 1).

### 2.4. Characterization

X-ray diffraction (XRD) measurements were performed on a PANalytical X’Pert Pro diffractometer (Malvern Panalytical, Kassel, Germany) with Cu Kα radiation (λ = 1.5406 Å) in Bragg–Brentano geometry. Data were collected over a 2θ range of 15–70° with a step size of 0.0263°. Raman spectra were collected using a Jobin Yvon T64000 triple-grating spectrometer (Horiba Jobin Yvon, Palaiseau, France) operating in subtractive mode and equipped with an N_2_–cooled CCD detector. Excitation was provided by the 514 nm line of an Ar^+^ ion laser, focused through an Olympus microscope objective with a 20.5 mm focal distance and a numerical aperture of 0.35. Each spectrum corresponds to the average of five accumulations with an integration time of 60 s. Surface morphology and the nanoparticle distribution were examined via field emission scanning electron microscopy (FESEM) using a JEOL JSM-6701F microscope (JEOL, Tokyo, Japan) operated at 7 kV. Nitrogen adsorption–desorption measurements were carried out using a Micromeritics ASAP 2020 (Micromeritics, Norcross, GA, USA) surface area and porosity analyzer at −196 °C, and the specific surface area was calculated using the Brunauer–Emmett–Teller (BET) method. Thermogravimetric analysis (TGA) was performed using a TGA Q500 thermal analyzer (TA Instruments, San Jose, CA, USA). Simultaneous thermogravimetric (TG) and differential scanning calorimetry (DSC) measurements were carried out using a simultaneous thermal analyzer (SDT 650, TA Instruments, San Jose, CA, USA). All experiments were conducted in air at a heating rate of 10 °C min^−1^ from room temperature to 1000 °C.

## 3. Results and Discussion

### 3.1. Structure Characterization

**Figure 2:**
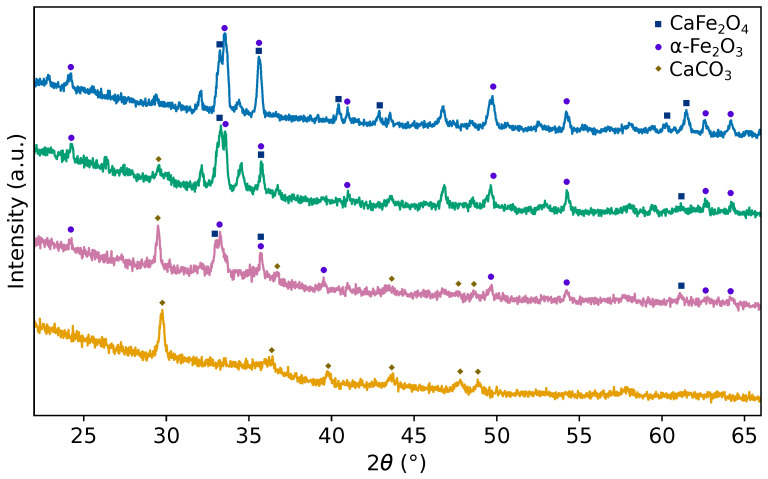
X-ray diffraction patterns of samples calcined at 550 (orange), 650 (reddish purple), 750 (bluish green), and 850 °C (blue).

The X-ray diffraction patterns of the samples calcined at 550, 650, 750, and 850 °C are shown in Figure 2. The diffraction patterns were analyzed by Rietveld refinement [27], and the refined structural parameters are summarized in Table 1. At 550 °C, the diffraction pattern is dominated by the characteristic reflections of the CaCO_3_ phase (COD 1547350) with space group $R\bar{3}c$, in agreement with previous reports [28], confirming the single-phase nature of the sample. The main reflections are located at 29.68°, 36.41°, 39.87°, 43.69°, 47.92°, and 49.02°, corresponding to the (104), (110), (113), (202), (10$\bar{8}$), and (116) planes, respectively.

Upon increasing the calcination temperature to 650 °C, the CaCO_3_ phase exhibits a slight expansion of the crystal lattice, with the unit cell volume increasing from 356.54 to 363.34 Å^3^, as obtained from the Rietveld refinement. At this temperature, reflections associated with the α-Fe_2_O_3_ phase ($R\bar{3}c$, COD 1532119) begin to appear, together with weak reflections of orthorhombic CaFe_2_O_4_ (*Pnma*, JCPDS 32-0168) at 33.01° and 35.74°. This behavior indicates the onset of CaFe_2_O_4_ formation during the decomposition of CaCO_3_. The coexistence of α-Fe_2_O_3_ and CaFe_2_O_4_ suggests a transient mixed-phase regime, which has been commonly observed in Ca–Fe oxide systems [29]. In addition, phase diagram analyses of the CaO–Fe_2_O_3_ system reported in the literature [16] indicate the existence of intermediate compositional and thermal regimes in which the simultaneous stabilization of multiple oxide phases is thermodynamically allowed.

**Figure 3:**
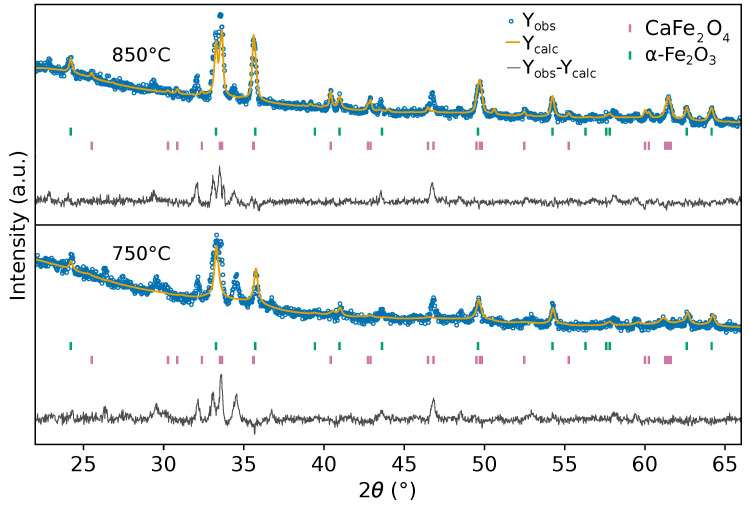
Rietveld refinement plots of the XRD patterns for samples calcined at 750 and 850 °C.

For the samples treated at 750 and 850 °C (Figure 3), the diffraction patterns are predominantly composed of reflections corresponding to the CaFe_2_O_4_ and α-Fe_2_O_3_ phases, suggesting the advanced progression of phase formation. The structural evolution is reflected in the changes in crystallite size, microstrain, and phase fractions obtained from the Rietveld analysis. The presence of weak, unindexed reflections at 34.12° and 34.56° is not attributed to the formation of additional crystalline phases, but is more likely associated with lattice imperfections or structural disorder. Similar weak features have been reported during the crystallization of CaFe_2_O_4_ and tend to disappear upon further thermal treatment as crystallinity increases [30].

**Figure 4:**
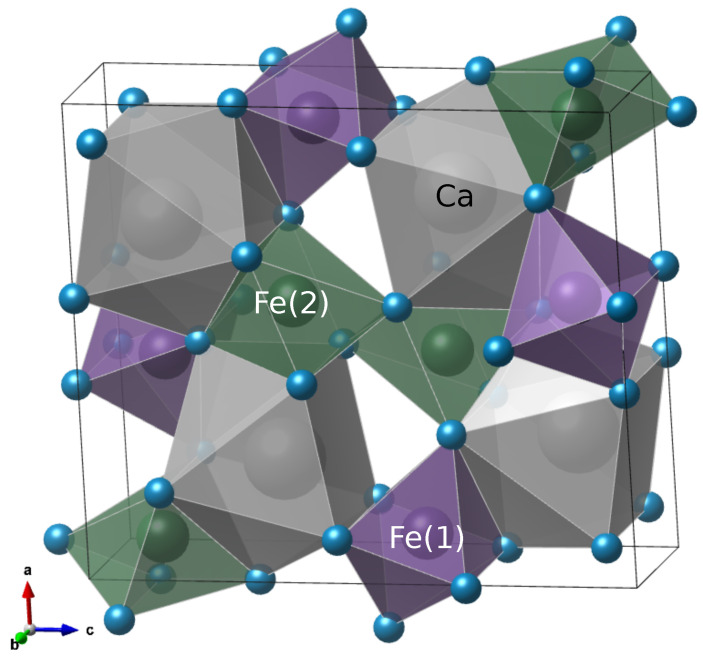
Orthorhombic *Pnma* structure of CaFe_2_O_4_ with two crystallographically distinct FeO_6_ octahedra. Drawn using VESTA 3.90.3a [31].

Table 2 presents the Fe–O bond lengths corresponding to the two octahedra Fe(1)O_6_ and Fe(2)O_6_ for the samples treated at 750 and 850 °C. At 750 °C, both octahedra display a wider distribution of Fe–O bond lengths (approximately 1.98 to 2.04 Å), which may be associated with residual lattice strain and local structural disorder. In contrast, the sample treated at 850 °C shows a narrower distribution of Fe–O distances (1.99 to 2.03 Å), consistent with the lower microstrain obtained from the Rietveld refinement. This decrease in bond length dispersion suggests a partial relaxation of the FeO_6_ framework, in agreement with the improvement in crystalline order at higher calcination temperatures. The Fe–O bond lengths are in good agreement with values reported for orthorhombic CaFe_2_O_4_ in the literature [12]. Figure 4 schematically illustrates the orthorhombic *Pnma* structure of CaFe_2_O_4_, highlighting the two crystallographically distinct FeO_6_ octahedra.

### 3.2. Morphological Characterization (FESEM)

**Figure 5:**
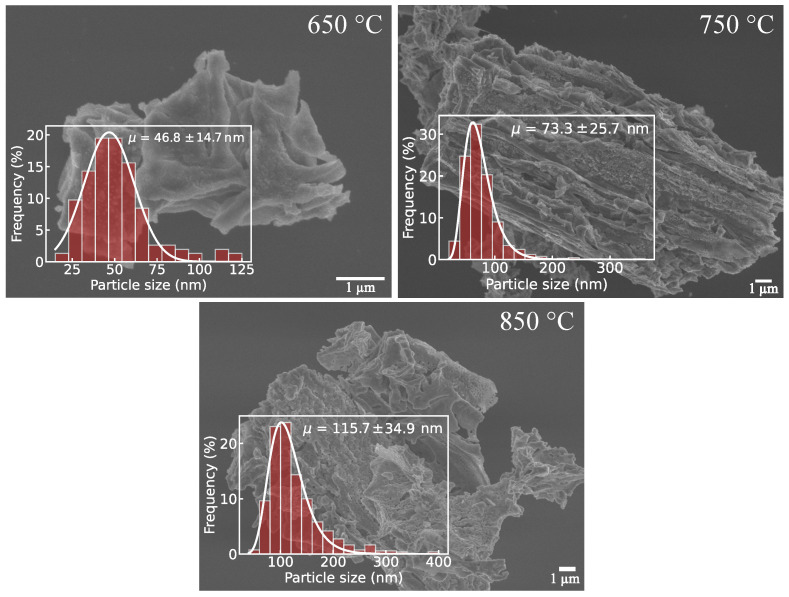
FESEM micrographs of CaFe_2_O_4_ powders calcined at 650, 750, and 850 °C. Insets show the corresponding particle size distributions.

The morphological features of the CaFe_2_O_4_ powders calcined at different temperatures were investigated by field-emission scanning electron microscopy (FESEM). Figure 5 shows representative micrographs of the samples calcined at 650, 750, and 850 °C, together with the corresponding particle size distributions measured using ImageJ 1.54g [32].

At all calcination temperatures, the material exhibits an aggregated morphology composed of irregularly shaped particles forming compact clusters. Occasional quasi-spherical agglomerates can be observed. However, the overall microstructure remains highly irregular, without well-defined faceted geometries or anisotropic features. This observation suggests that particle growth proceeds predominantly through non-directional coalescence mechanisms. Similar aggregation-dominated morphologies have been reported for CaFe_2_O_4_ and related ferrite oxides synthesized by wet chemical and polymeric precursor routes, where calcination in air promotes particle coalescence and grain growth [15,33].

A clear evolution in particle size with calcination temperature is observed. For the sample calcined at 650 °C, the particle size distribution is centered at μ=46.8±14.7 nm, indicating the presence of nanoscale particles with a relatively narrow size distribution. The corresponding FESEM micrograph reveals loosely aggregated particles, suggesting limited particle coalescence at this temperature. Upon increasing the calcination temperature to 750 °C, the average particle size increases to μ=73.3±25.7 nm, accompanied by a broader size distribution. This behavior suggests enhanced particle coalescence and grain growth at higher temperatures. At 850 °C, the particle size further increases to μ=115.7±34.9 nm, with a tail toward larger sizes, indicating significant grain growth and partial sintering processes.

The systematic increase in both the average particle size and the width of the size distribution with increasing calcination temperature is consistent with thermally induced coarsening. At higher temperatures, atomic diffusion may facilitate the coalescence of neighboring particles, leading to larger aggregates. Similar temperature-dependent particle growth trends have been widely reported for CaFe_2_O_4_ and related ferrite systems [34,35].

From a microstructural standpoint, the particle growth observed with increasing calcination temperature is consistent with progressive densification and reduction in the accessible surface area. This interpretation is further supported by the specific surface area values obtained from nitrogen physisorption (BET) measurements, which indicate a low surface area characteristic of CaFe_2_O_4_ treated at high temperature. The morphological evolution revealed by FESEM therefore reflects grain growth and possible sintering during calcination in air.

### 3.3. Optical Properties

**Figure 6:**
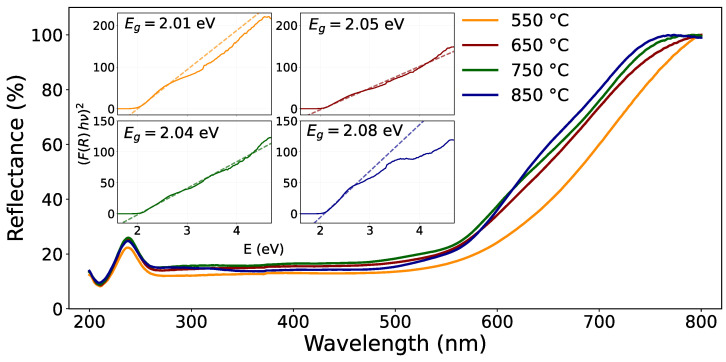
UV-Vis diffuse reflectance spectra of CaFe_2_O_4_ samples calcined at different temperatures. The inset shows the corresponding Kubelka–Munk–based Tauc plots assuming direct allowed transitions, which were used to estimate the optical band gap energy ($E_{g}$) for each sample by linear extrapolation (dashed lines).

The UV-Vis diffuse reflectance spectra (Figure 6) were analyzed using the Kubelka–Munk formalism, in which the reflectance data are transformed into the function F(R), proportional to the optical absorption coefficient under ideal diffuse scattering conditions [36]. The optical band gap energy ($E_{g}$) was estimated using the Tauc method, according to the following relation [37]:

(1) $$F\left(R\right)\;h\nu =A\;{\left(h\nu -E_{g}\right)}^{n},$$

where *A* is a proportionality constant, hν is the photon energy, and *n* depends on the type of the electronic transition. In the present analysis, a direct allowed transition was assumed (n=2). Accordingly, plots of ${\left(F\left(R\right)\;h\nu \right)}^{2}$ versus hν were constructed, and the optical band gap energy $E_{g}$ was determined by extrapolating the linear region of the Tauc plot to the energy axis [37].

The diffuse reflectance spectra of CaFe_2_O_4_ samples calcined at different temperatures exhibit a smooth and monotonic spectral behavior over the analyzed wavelength range, without the appearance of additional absorption bands or abrupt changes. All samples display very similar spectral profiles, with no significant modification in the overall shape of the reflectance curves as a function of calcination temperature. This indicates that the optical absorption characteristics of CaFe_2_O_4_ are preserved across the investigated thermal treatments. Small variations in reflectance intensity can be attributed to differences in microstructure or surface morphology induced by calcination, rather than to the formation of new electronic transitions.

The optical band gap energies ($E_{g}$), calculated from the Kubelka–Munk function using Tauc plots, lie in the range of 2.01–2.08 eV for the samples calcined at 550, 650, 750, and 850 °C.

The band gap energies obtained in this work are close to ∼2.0 eV, consistent with previously reported values for CaFe_2_O_4_ prepared using different synthesis routes. For instance, Bouatama et al. reported a direct allowed band gap of 1.83 eV for sol–gel derived CaFe_2_O_4_, attributed to crystal-field transitions of octahedrally coordinated Fe^3+^ ions [38]. Dom et al. showed that CaFe_2_O_4_ powders prepared via solid-state reaction, polymeric precursor, microwave, and sol–gel combustion methods exhibit band gap values in the narrow range of 1.83–1.92 eV, suggesting that the optical band gap is only weakly affected by the synthesis method [39].

Band gap values in this energy range lie within the visible region of the electromagnetic spectrum, enabling absorption of visible light and generation of photoinduced charge carriers, consistent with the observed photo-assisted activity. Previous studies have shown that compositional modifications, such as transition-metal doping or hybridization with carbon-based matrices, can induce measurable shifts in the band gap [40]. This suggests that changes in the band gap of CaFe_2_O_4_ systems depend more on chemical composition than on thermal treatment.

### 3.4. Fourier-Transform Infrared (FT-IR) Spectroscopy

**Figure 7:**
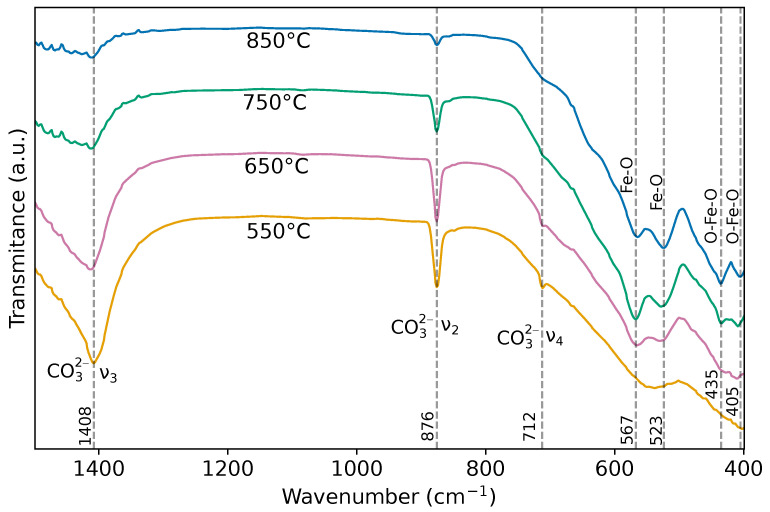
FT-IR spectra of the samples calcined at 550, 650, 750, and 850 °C.

The FT-IR spectra in Figure 7 exhibit a progressive evolution from a chemically heterogeneous system toward a structurally consolidated orthorhombic CaFe_2_O_4_ lattice, where two main groups of bands can be identified: (i) residual carbonate species and (ii) the inorganic Fe–O network based on FeO_6_ units. The intense bands located at ∼1408 cm^−1^ ($\nu_{3}$), ∼871 cm^−1^ ($\nu_{2}$), and ∼712 cm^−1^ ($\nu_{4}$) are characteristic of the internal modes of the ${\mathrm{CO}}_{3}^{2-}$ anion [41,42], evidencing the presence of surface or interstitial calcium carbonate, particularly dominant in the sample calcined at 550 °C. The progressive attenuation of these bands upon increasing the temperature to 750–850 °C suggests an interfacial chemical reorganization process rather than a simple thermal decomposition of CaCO_3_, where the carbonate acts transiently as a Ca^2+^ reservoir and is gradually consumed in the stabilization of the calcium ferrite. This behavior reveals a scenario in which the disappearance of the carbonate signals is simultaneous with the strengthening of the CaFe_2_O_4_ framework, consistent with XRD results.

The region between ∼400 and 650 cm^−1^ is dominated by Fe–O lattice vibrations [43], characteristic of FeO_6_ octahedra, which constitute the main structural units of the orthorhombic CaFe_2_O_4_ phase. The bands at ∼407–434 cm^−1^ are assigned to O–Fe–O bending modes. Their broad presence at low temperatures indicates a more disordered local environment. As the calcination temperature increases, the band at ∼521 cm^−1^ emerges with greater definition, associated with intermediate-energy Fe–O stretching, whose progressive growth constitutes a direct spectroscopic signature of the increase in vibrational coherence and the collapse of the distribution of Fe–O force constants. This phenomenon reflects a substantial reduction in structural disorder and a homogenization of the octahedral environment. Any residual contribution of α-Fe_2_O_3_ may overlap with Fe–O modes, explaining its spectral invisibility in FT-IR despite potential diffraction evidence. Finally, the absence of Ca–O bands in the measured spectral range is consistent with the fact that these vibrations, being highly ionic and low-energy in nature, are located below 400 cm^−1^ [41], outside the instrumental window. Overall, Figure 7 supports the formation of CaFe_2_O_4_ and shows the vibrational changes driven by calcination.

### 3.5. Raman Spectroscopy

**Figure 8:**
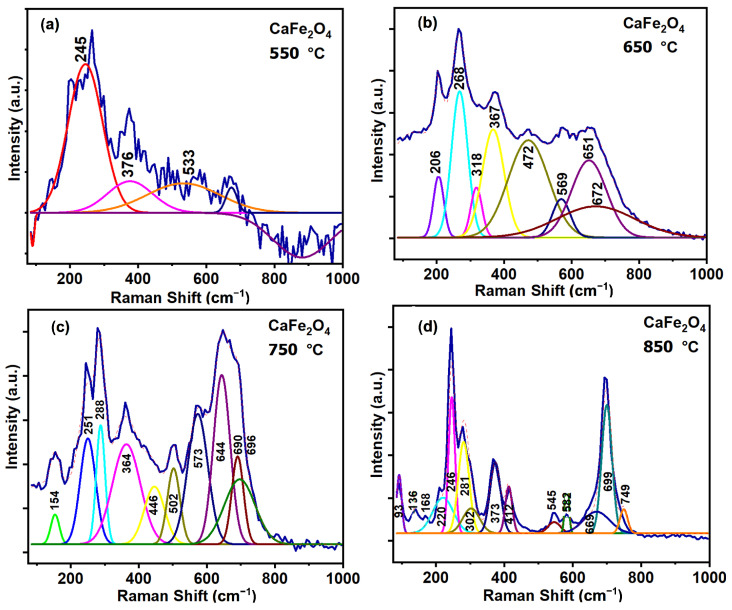
Raman spectra recorded at room temperature using a 514 nm excitation wavelength for CaFe_2_O_4_ samples calcined at (**a**) 550, (**b**) 650, (**c**) 750, and (**d**) 850 °C.

CaFe_2_O_4_ crystallizes in an orthorhombic unit cell whose primitive cell gives rise to 84 optical phonon modes, of which 42 are Raman active (14A_*g*_ + 7B_1*g*_ + 14B_2*g*_ + 7B_3*g*_) [44]. Raman spectra were collected at room temperature from powdered samples without polarization control, and therefore represent an orientational average over randomly oriented crystallites [44,45]. Under these conditions, all Raman-active symmetry species contribute simultaneously to the measured response, resulting in intrinsically broadened and partially overlapping bands [46]. Excitation at λ=514 nm selectively enhances specific Fe-O vibrational modes due to pre-resonance effects associated with electronic transitions of Fe^3+^ ions, increasing the sensitivity of Raman spectroscopy to local distortions of the FeO_6_ octahedra and to the distribution of crystalline domain sizes [47]. In this context, the spectrum recorded at 550 °C , Figure 8a is characterized by broad and poorly resolved bands, a dominant maximum near ∼245 cm^−1^, weak contributions around ∼376 and ∼533 cm^−1^, and an elevated background, reflecting a high degree of structural disorder and incomplete lattice consolidation.

In Figure 8b, the Raman spectrum recorded at 650 °C evidences a clear evolution toward increased structural organization, as indicated by the emergence of better-defined bands around ∼206, 268, 318, and 367 cm^−1^. These features mark the onset of FeO_6_ octahedral ordering and a progressive enhancement in vibrational coherence within the lattice. At this intermediate thermal stage, the spectrum reflects a local coexistence of structural contributions: bands in the ∼250–350 cm^−1^ range and above ∼500 cm^−1^ are consistent with the incipient formation of CaFe_2_O_4_, while the persistence of broad spectral components points to residual disorder and incompletely transformed Ca–Fe–O environments [48].

At 750 °C (Figure 8c), the Raman spectrum becomes markedly more structured, displaying sharp and reproducible bands that signal the consolidation of the orthorhombic CaFe_2_O_4_ lattice. Well-defined lattice modes appear in the ∼251–288 cm^−1^ region, accompanied by bands at ∼364, 446, and 502 cm^−1^ associated with FeO_6_ octahedral deformations. In the high-frequency region, the development of intense bands at ∼573, 644, and 690–696 cm^−1^ is assigned to Fe–O stretching modes, indicating increased bond stiffness and internal structural order. Under these conditions, the Raman response is dominated by Fe-O-Fe bending and Fe-O stretching vibrations characteristic of a stabilized CaFe_2_O_4_ crystalline framework [44].

A quantitative analysis of the individual vibrational modes was performed using the experimental Raman spectra of CaFe_2_O_4_ by fitting the Raman bands with Lorentzian line shapes. For each vibrational mode *i*, the spectral position $\omega_{i}$, the full width at half maximum (FWHM) $\Gamma_{i}$, and the integrated area $A_{i}$ were extracted, where $\omega_{i}$ corresponds to the mode frequency (cm^−1^), $\Gamma_{i}$ represents the linewidth (cm^−1^), and $A_{i}$ denotes the integrated band intensity (arbitrary units).

The Raman linewidth $\Gamma_{i}$ is directly related to the phonon lifetime $\tau_{i}$, which reflects phonon-phonon scattering processes as well as the influence of structural defects and local disorder [48]. Under the commonly adopted assumption of exponential phonon damping, the phonon lifetime can be estimated from the FWHM according to

$$\tau_{i}\approx \frac{1}{2\pi c\;\Gamma_{i}}$$

where $c=2.998\times 10^{10}$ cm s^−1^ is the speed of light. This relation follows from the Fourier transform of a damped harmonic oscillation and is widely employed in the Raman analysis of crystalline oxides.

Accordingly, a reduction in $\Gamma_{i}$ (Raman band narrowing) corresponds to an increase in $\tau_{i}$, indicating enhanced vibrational coherence and a suppression of scattering mechanisms. This behavior is fully consistent with the progressive structural consolidation and increased local order of the orthorhombic CaFe_2_O_4_ lattice.

As summarized in Table 3, the phonon lifetimes extracted from the Raman linewidths fall within the characteristic range reported for polycrystalline oxides, indicating a damping regime governed by multiple scattering mechanisms. These include phonon scattering at grain boundaries, finite crystallite size effects, microstrain, and local distortions of the FeO_6_ polyhedra, with additional contributions arising from point defects and intrinsic anharmonic phonon-phonon coupling at elevated calcination temperatures [47]. The magnitude of the phonon lifetimes therefore provides a sensitive measure of the local structural order of the material. In this context, the observed increase in phonon lifetime with increasing calcination temperature suggests a progressive reduction in local structural disorder and an enhancement in vibrational coherence within the CaFe_2_O_4_ lattice [45]. To further quantify the degree of local structural order, a global Raman order parameter, $Q_{\mathrm{Raman}}$, was evaluated. This parameter provides a measure of the average vibrational coherence of the crystalline lattice and therefore reflects the extent of local structural ordering. The global Raman order index is defined as

$$Q_{\mathrm{Raman}}=\frac{1}{N}\sum_{i=1}^{N}\frac{w_{i}}{\Gamma_{i}},$$

where *N* is the total number of Raman-active modes considered, $w_{i}$ denotes the weighting factor associated with the *i*-th vibrational mode (typically proportional to its integrated intensity or relative spectral contribution), and $\Gamma_{i}$ is the corresponding full width at half maximum (FWHM).

$$Q_{\mathrm{Raman}}\approx 15.00.$$

This value can be interpreted as an average vibrational quality factor, representative of a well consolidated polycrystalline lattice with moderate to high phonon coherence and residual disorder associated with finite domain size, distortions of the FeO_6_ octahedra, and grain boundary effects.

The increase in $Q_{\mathrm{Raman}}$ reported in Table 4 with increasing calcination temperature reflects a progressive enhancement in phonon coherence and short-range structural order in CaFe_2_O_4_, culminating in a highly consolidated crystalline lattice at 850 °C [44]. The hematite contribution at 850 °C is negligible, as its Raman-active modes largely overlap with those of CaFe_2_O_4_, and XRD detects it only as a trace phase.

### 3.6. Textural Characterization

**Figure 9:**
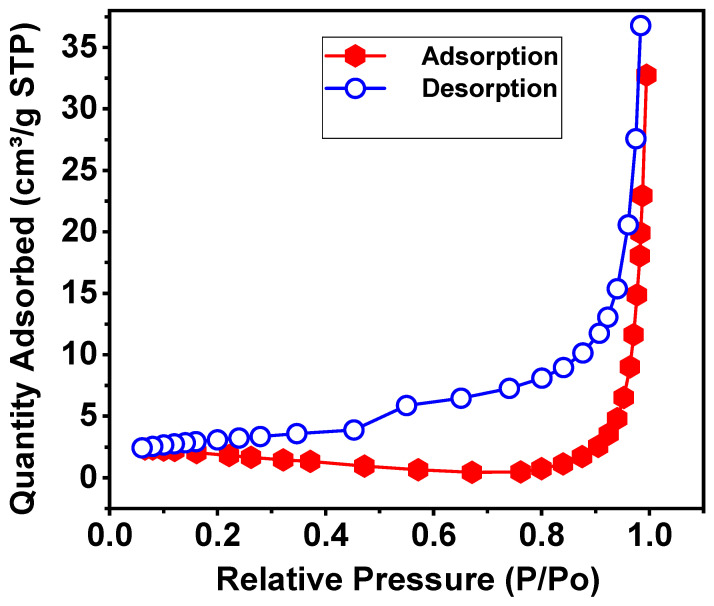
N_2_ adsorption–desorption isotherms of CaFe_2_O_4_ calcined at 750 °C.

Figure 9 shows the N_2_ adsorption–desorption isotherms measured at 77 K for the CaFe_2_O_4_ sample calcined at 750 °C. The isotherm exhibits low N_2_ uptake at low relative pressures ($P/P_{0}<0.3$), followed by a gradual increase in the intermediate pressure region and a sharp rise in the adsorbed volume at high relative pressures ($P/P_{0}>0.8$), accompanied by a wide hysteresis loop between the adsorption and desorption branches.

In the region with low relative pressure, the adsorbed amount is small and increases slowly with $P/P_{0}$, indicating a low density of high-energy adsorption sites and the absence of significant microporosity. This behavior is consistent with the moderate BET surface area value (∼5–6 m^2^ g^−1^) and reflects the dense and crystalline nature of orthorhombic CaFe_2_O_4_ after high-temperature calcination. The lack of a pronounced uptake in this region confirms that the initial adsorption mainly occurs on weakly defective external surfaces. In the intermediate pressure region, adsorption increases gradually, which is associated with the formation of adsorbed layers on the external surface and the progressive filling of interparticle voids. The moderate slope indicates a limited contribution from well-defined mesopores, confirming that the porosity is predominantly textural and not intrinsic to the crystal lattice [49].

The abrupt increase in adsorbed volume at high relative pressures $\left(P/P_{0}\to 1\right)$ is indicative of capillary condensation in wide mesopores and macropores associated with interparticle voids, reflecting that adsorption is dominated by large accessible spaces rather than a high surface area. The wide hysteresis between the adsorption and desorption branches reveals a complex and irreversible porous connectivity, characteristic of interparticle porosity with narrow necks and ink-bottle effects, confirming that the material’s texture is dominated by the architecture of the agglomerates and not by a uniform mesoporous network [49].

In other words, from a physical standpoint, Figure 9 reveals that CaFe_2_O_4_ calcined at 750 °C is a thermally consolidated oxide with a low surface area, porosity dominated by interparticle voids, and minimal microporosity. This explains the absence of bactericidal activity by diffusion, due to the limited surface–bacteria interaction and the lack of diffusible species. It exhibits moderate photocatalysis, controlled by interfacial processes under irradiation rather than by a high active area. Thus, its high chemical stability is advantageous for environmental applications where leaching or uncontrolled toxicity must be avoided. Adsorption is dominated by capillary condensation in large textural pores, typical of oxides calcined at high temperature, giving CaFe_2_O_4_ 750 °C a robust structure with porosity accessible for mass transport but low surface reactivity.

According to the IUPAC classification, the N_2_ adsorption–desorption isotherms of the CaFe_2_O_4_ material calcined at 750 °C (Figure 9) can be assigned to a type IV isotherm, characteristic of mesoporous solids. This isotherm exhibits a wide hysteresis loop at high relative pressures, associated with capillary condensation phenomena occurring in pores larger than the microporous range. In a conservative interpretation, the hysteresis loop can be described as type H3, typically associated with non-rigid aggregates of lamellar or irregular particles and with porosity dominated by interparticle voids rather than well-defined cylindrical mesopores [49].

**Figure 10:**
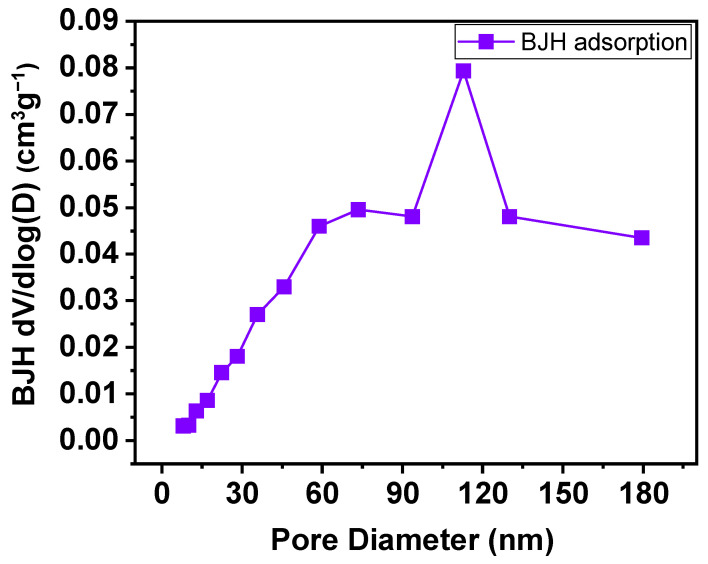
BJH pore size distribution derived from the adsorption branch for CaFe_2_O_4_ calcined at 750 °C.

This classification is fully consistent with the BJH pore size distribution obtained from the adsorption branch (Figure 10), which reveals a broad distribution centered in the meso-macroporous range, with a dominant contribution from pore diameters greater than several tens of nanometers. The absence of a pronounced maximum in the small mesopore region and the prevalence of large pores support the interpretation that the observed porosity originates primarily from textural effects associated with particle packing and agglomeration. Consequently, the BJH analysis confirms that the porous system of CaFe_2_O_4_ is not intrinsic to the crystal lattice, but is governed by an interparticle meso-macroporosity, consistent with the shape of the adsorption–desorption isotherm and the large hysteresis loop observed near $\left(P/P_{0}\to 1\right)$ [50].

N_2_ physisorption analysis (Table 5) indicates that CaFe_2_O_4_ calcined at 750 °C exhibits a modest specific surface area ($S_{\mathrm{BET}}=5.575\pm 0.383$ m^2^ g^−1^), consistent with a thermally consolidated calcium ferrite. The total pore volume estimated at high relative pressure ($P/P_{0}\approx 0.98$) is $V_{t}=0.03080$ cm^3^ g^−1^, while BJH analysis from the adsorption branch yields $V_{\mathrm{BJH}}=0.05064$ cm^3^ g^−1^, with a mean pore diameter of $D_{\mathrm{BJH}}=60.85$ nm. Overall, the textural parameters support a pore system dominated by interparticle meso–macroporosity, in agreement with the adsorption–desorption isotherm and the BJH pore size distribution.

### 3.7. Thermal Analysis

**Figure 11:**
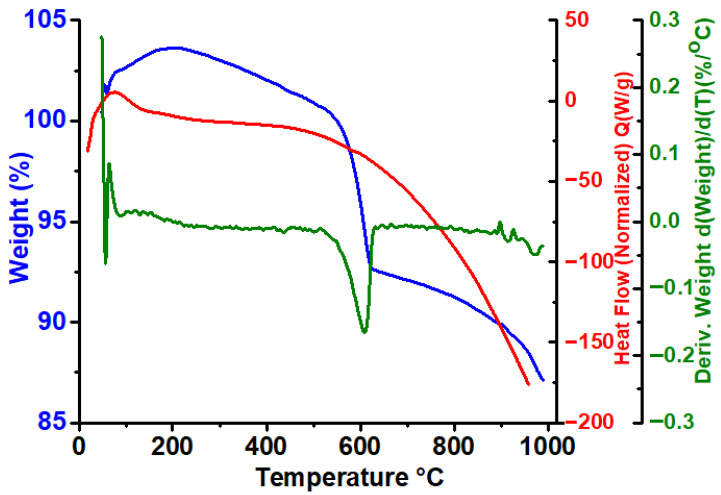
Thermogravimetric (TG), derivative thermogravimetric (DTG), and differential scanning calorimetry (DSC) curves of CaFe_2_O_4_ calcined at 750 °C, recorded in air at a heating rate of 10 °C min^−1^ up to 1000 °C.

Figure 11 presents the TG, DTG, and DSC profiles of CaFe_2_O_4_ calcined at 750 °C, measured in air up to 1000 °C. The thermogravimetric curve shows a nearly constant mass in the low- and intermediate-temperature regions, indicating the absence of physically adsorbed water, residual solvents, or unreacted organic species [51,52]. This behavior suggests the effective removal of precursors during the calcination step and the formation of a thermally stable oxide, as also indicated by XRD results.

A small mass variation below 150 °C, accompanied by a weak DTG signal, may be associated with baseline fluctuations or the release of trace surface-adsorbed species, and does not significantly affect the material stability. In the temperature range from approximately 150 to 550 °C, no significant mass loss or thermal events are detected, indicating the high thermal robustness of the orthorhombic CaFe_2_O_4_ structure [51].

A distinct DTG minimum and a corresponding exothermic DSC feature are observed in the range of 580–650 °C, associated with a moderate mass loss. This event is unlikely to be related to phase decomposition, but may be associated with structural rearrangements occurring and the decomposition of residual carbonate species. The latter is consistent with FT-IR observations, which evidenced characteristic ${\mathrm{CO}}_{3}^{2-}$ vibrations in samples calcined at 750 °C. Furthermore, this thermal event could involve processes such as structural relaxation or crystallinity enhancement. Although TG–DSC alone does not allow direct identification of defect-related mechanisms, such thermal relaxation phenomena are consistent with behaviors commonly reported for ferrites subjected to high-temperature treatments [53].

At higher temperatures (>700 °C), the TG curve shows a gradual and continuous mass decrease without sharp DTG or DSC features, which can be attributed to slow oxygen loss or high-temperature lattice diffusion processes. No significant thermal decomposition is observed up to 1000 °C, indicating the high thermal stability of CaFe_2_O_4_ under oxidizing conditions [53].

### 3.8. Photo-Assisted Removal and Antibacterial Evaluation

Ciprofloxacin was quantified using a 1260 Infinity II LC high-performance liquid chromatograph (Agilent, Santa Clara, CA, USA) equipped with a UV-Vis (277 nm) detector, and a Poroshell 120 C-18 column (4.6 × 100 mm, 4 μm). The flow rate was set at 0.9 mL min^−1^, and the mobile phase was composed of Formic Acid (FA) 0.001% (*v v*^−1^)/ Acetonitrile (ACN) in gradient mode. The gradient program was as follows: 0 min, 95% FA/ 5% ACN; 0–4 min, 80% FA/ 20% ACN; 4–6 min, 60% FA/ 40% ACN; 6–10 min, 20% FA/ 80% ACN; 10–12 min, 50% FA/ 50% ACN; 12–15 min, 95% FA/ 5% ACN. The column temperature was maintained at 40 °C, the injection volume was 10 μL, and detection was performed at 277 nm.

**Figure 12:**
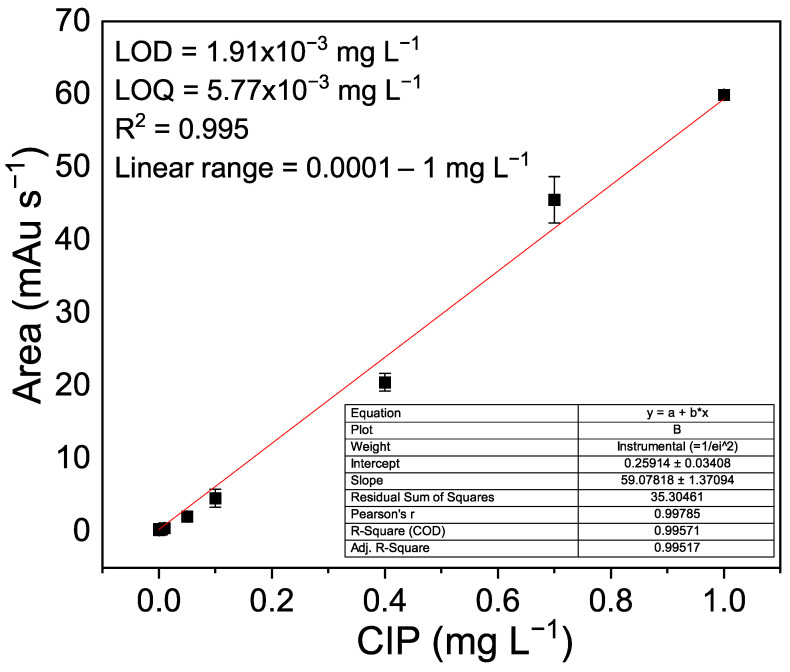
Calibration curve for CIP quantification obtained via HPLC with UV-Vis detection (277 nm).

For CIP quantification, a calibration curve was performed (Figure 12). The linear range varied from 0.0001 to 1 mg L^−1^, the retention time of CIP = 5.64 min, LOD = 1.91 × 10^−3^ mg L^−1^, LOQ = 5.77 × 10^−3^ mg L^−1^, and ^R2^ = 0.995. Under UV-A irradiation, CaFe_2_O_4_ can generate electron–hole pairs due to its narrow band gap (≈1.9–2.1 eV). The photogenerated electrons may reduce dissolved oxygen to superoxide radicals ($O_{2}^{•-}$),while the holes can oxidize water or surface hydroxyl groups to produce hydroxyl radicals (•OH). These reactive oxygen species are commonly considered the main oxidative agents responsible for the degradation of organic molecules such as ciprofloxacin on the catalyst surface.

**Figure 13:**
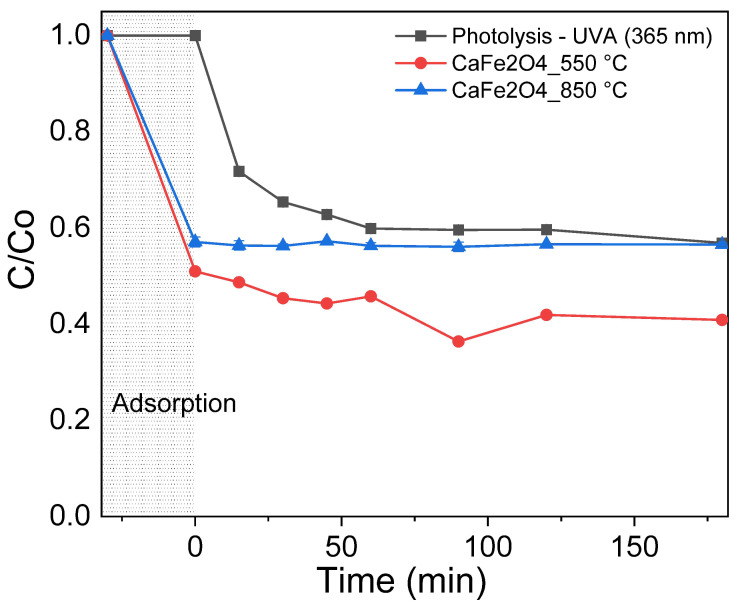
Degradation profile of CIP under UV-A light (365 nm).

Figure 13 shows the removal profile of ciprofloxacin (CIP) as a function of time under UV-A irradiation. After 30 min in the dark, CIP removal reached 43% for CaFe_2_O_4_ calcined at 850 °C and 49% for CaFe_2_O_4_ calcined at 550 °C, indicating that adsorption of the drug onto the surface of the materials plays a significant role in the removal process. These results indicate that ciprofloxacin removal under the present conditions is mainly associated with adsorption, with irradiation providing an additional contribution. This behavior reflects a balance between crystallinity and surface accessibility, which ultimately governs the photo-assisted response. Under UV-A irradiation, no further CIP removal was observed for the CaFe_2_O_4_ 850 °C sample. In contrast, the sample calcined at 550 °C exhibited enhanced activity, with total removal reaching 59%, corresponding to an additional 10% after the adsorption stage. The photocatalytic behavior observed for CaFe_2_O_4_ cannot be adequately described by a single kinetic model, as it arises from the interplay between surface adsorption, structural consolidation, and charge transfer processes. In particular, the apparent deviation from ideal pseudo-first-order kinetics and the tendency toward zero-order behavior at higher calcination temperatures suggest a regime dominated by surface saturation and limited availability of active sites [54,55]. This interpretation is consistent with previous studies emphasizing that photocatalytic degradation is often governed by adsorption–desorption equilibria and surface-mediated mechanisms rather than intrinsic reaction order [55,56]. Moreover, recent analyses have highlighted that the widespread use of simplified pseudo-first-order models can be misleading, as the observed kinetics strongly depend on experimental conditions, catalyst surface properties, and pollutant concentration [54,57]. In this context, the reduced activity of highly calcined CaFe_2_O_4_ can be attributed to enhanced crystallinity accompanied by a loss of surface accessibility, which limits both adsorption capacity and interfacial charge transfer [56]. Overall, these findings support the view that photocatalytic kinetics should be interpreted within a comprehensive framework that accounts for light absorption, surface coverage, and mechanistic complexity, rather than relying on simplified rate laws [54,57].

To contextualize the observed ciprofloxacin removal efficiency, it is important to note that many studies on pharmaceutical degradation rely on composite materials, heterostructures, or Z-scheme systems designed to enhance charge separation and overall removal efficiency [1,2,15]. In contrast, studies addressing the degradation of ciprofloxacin using phase-pure CaFe_2_O_4_ without additional semiconductors or surface modification are limited. Within this framework, the partial removal of ciprofloxacin observed in the present work indicates that structurally consolidated CaFe_2_O_4_ can participate in photo-assisted processes under UV-A irradiation, even in the absence of engineered heterojunctions. Although the overall removal efficiency is moderate and largely influenced by adsorption, these results suggest that CaFe_2_O_4_ participates in photo-assisted interfacial processes while preserving high chemical stability.

It is worth noting that, at intermediate and high calcination temperatures, the material behaves as a multiphase system in which the CaFe_2_O_4_ framework coexists with secondary crystalline phases, such as α-Fe_2_O_3_, as indicated by XRD results. Raman spectroscopy confirms the progressive establishment of the orthorhombic CaFe_2_O_4_ structure (*Pnma* symmetry), indicating that adsorption and photo-assisted processes arise from an evolving and structurally consolidating calcium ferrite matrix rather than from a strictly single-phase material.

The photoactivity of CaFe_2_O_4_ toward methylene blue degradation has been previously reported in the literature [58]. In the present study, ciprofloxacin, a more recalcitrant organic contaminant, was used as a target molecule, and removal efficiencies of up to 59% were obtained under the investigated conditions. It is worth noting that the removal performance of CaFe_2_O_4_ can be further enhanced through appropriate doping strategies, as reported elsewhere [58], which will be explored in future work.

#### Antibacterial Activity Test

**Figure 14:**
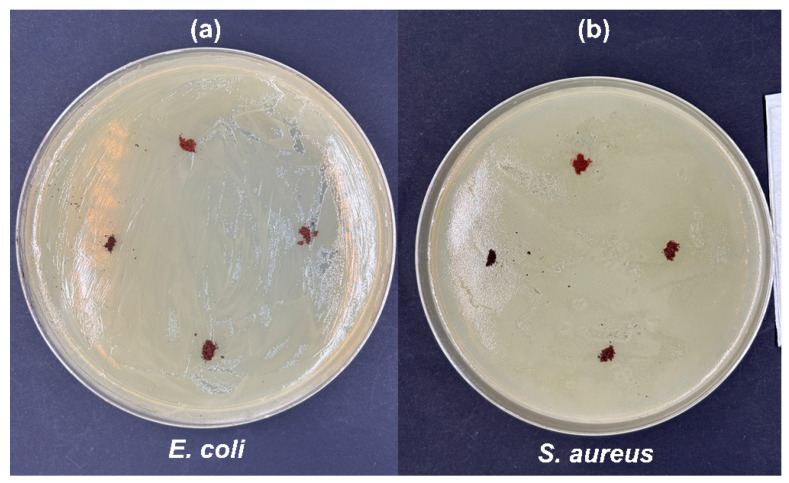
Inhibition zone of (**a**) *E. coli* and (**b**) *S. aureus* (at 550, 650, 750, and 850 °C) for CaFe_2_O_4_.

The CaFe_2_O_4_ samples calcined at 550, 650, 750, and 850 °C did not exhibit inhibition halos against either *E. coli* (Gram-negative) or *S. aureus* (Gram-positive) in the agar diffusion assay (Figure 14). Although this result may appear inconsistent with the photocatalytic activity observed under UV-A irradiation, it can be understood considering the structural nature of CaFe_2_O_4_, its textural properties, and the limitations of diffusion-based antibacterial tests.

A characteristic feature of the calcined CaFe_2_O_4_ powders is the low specific surface area. Across 550–850 °C, the BET surface area remains modest (typically in the ∼5–6 m^2^ g^−1^ range), which is common for orthorhombic calcium ferrites obtained after high-temperature calcination. Such values indicate a low density of accessible surface sites per unit mass. In addition, the porosity inferred from N_2_ adsorption–desorption measurements is mainly associated with interparticle voids rather than intrinsic micro- or mesoporosity. Textural porosity contributes to pore volume, but it does not necessarily translate into a high population of chemically active sites.

Agar diffusion assays are sensitive to the presence of diffusible inhibitory species. Many inorganic materials produce inhibition halos because they release ions (e.g., Ag^+^, Cu^2+^, Zn^2+^) or reactive soluble products that diffuse through the agar and inhibit bacterial growth at a distance [59]. In contrast, CaFe_2_O_4_ is a stable mixed oxide in which Ca^2+^ and Fe^3+^ are part of the crystal lattice, and significant ion release under near- neutral conduction is not expected [60]. Without sufficient release of soluble species, the formation of a clear inhibition halo becomes unlikely, which explains the full bacterial growth observed for both strains.

Calcination temperature strongly influences defect density, surface hydroxylation, and the fraction of undercoordinated metal sites [61]. As the temperature increases from 550 to 850 °C, crystallinity typically increases while the concentration of structural defects (e.g., oxygen vacancies, disordered surface layers, residual hydroxyls) decreases. Defects can act as reactive centers for redox processes or for generating interfacial oxidative stress in contact with biological media. Therefore, thermal consolidation is expected to make the surface less reactive and the solid more inert. This trend is consistent with the absence of a bactericidal response across the studied temperature range.

The photocatalytic degradation of ciprofloxacin under UV-A irradiation suggests that CaFe_2_O_4_ could potentially generate reactive oxygen species (ROS), such as •OH and $O_{2}^{•-}$, on its surface under illumination. However, such processes occur at the solid–liquid interface and require direct contact between the material and the target molecule. In an agar diffusion assay, the response depends on the diffusion of inhibitory species through the solid medium, rather than on localized interfacial reactions. Reactive oxygen species generated during photocatalysis are short-lived and do not diffuse over macroscopic distances in agar [62], which further explains the absence of inhibition halos.

Overall, the negative antibacterial result is consistent with the low surface area, limited ion release, and high structural stability of CaFe_2_O_4_. The absence of inhibition halos therefore does not contradict the photocatalytic behavior of the material but reflects the different mechanisms involved in diffusion-based antibacterial tests and irradiation-driven interfacial reactions. No direct ion-release or toxicity measurements were performed, and therefore conclusions regarding environmental or biological safety are limited to the experimental observations reported here.

## 4. Conclusions

The present study investigates the structural, vibrational, and functional behavior of CaFe_2_O_4_ synthesized via the Pechini polymeric precursor method. Multi-analytical characterization reveals a well-defined thermal evolution from a partially carbonated and structurally disordered precursor at 550 °C to a highly ordered orthorhombic lattice at 850 °C. Rietveld refinement confirms that the stabilization of the *Pnma* phase is accompanied by relaxation of the FeO_6_ octahedral framework, as indicated by the narrowing of Fe–O bond length distributions. This structural consolidation is further supported by FT-IR and Raman spectroscopies; notably, quantitative Raman analysis demonstrated a nearly three-fold increase in the global Raman order parameter ($Q_{\mathrm{Raman}}$) and phonon lifetimes, signaling a drastic enhancement in vibrational coherence and the effective suppression of lattice defects as the calcination temperature increases.

From a functional point of view, the results indicate that structural ordering and chemical stability do not necessarily lead to higher interfacial reactivity. The CaFe_2_O_4_ samples show a modest photo-assisted response during ciprofloxacin removal under UV-A irradiation, while no antibacterial activity is detected in agar diffusion assays against *E. coli* and *S. aureus*. The absence of inhibition halos suggests weak biological interaction within the sensitivity of the assay, which is consistent with the high chemical stability of the ferrite lattice and the modest specific surface area measured by N_2_ physisorption. However, no direct ion-release, dissolution, or toxicity measurements were performed; therefore, the discussion of environmental or biological safety is limited to the experimental observations reported in this work.

Overall, the combined structural, spectroscopic, textural, and functional results indicate that CaFe_2_O_4_ is a structurally robust and chemically stable ceramic material. Its ability to maintain surface reactivity under irradiation, together with the absence of bactericidal effects, suggests its suitability for environmental purposes like water remediation. Further studies addressing ion release, recycling capacity, and long-term environmental impact are necessary to fully assess its environmental safety.

## Figures and Tables

**Table 1:** Rietveld refinement parameters obtained from XRD data at different calcination temperatures.

Phase	Parameter	850 °C	750 °C	650 °C	550 °C
CaFe_2_O_4_ (*Pnma*)	*a* (Å)	9.2081(13)	9.342(28)	9.438(5)	–
	*b* (Å)	3.0081(4)	2.983(4)	2.9743(19)	–
	*c* (Å)	10.6847(19)	10.66(3)	10.691(7)	–
	Volume (Å^3^)	295.96(6)	297.1(4)	300.15(23)	–
	Size (nm)	74(3)	35(6)	67(12)	–
	Microstrain (%)	0.105(27)	0.85(21)	0.19(12)	–
	Wt. fraction	0.584(8)	0.562(26)	0.265(19)	–
Fe_2_O_3_ ($R\bar{3}c$)	*a* (Å)	5.0237(14)	5.0187(22)	5.0205(23)	–
	*c* (Å)	13.7021(29)	13.700(5)	13.709(5)	–
	Volume (Å^3^)	299.48(9)	298.84(15)	299.23(14)	–
	Size (nm)	46.6(17)	42.1(26)	53(3)	–
	Microstrain (%)	0.08(3)	0.04(5)	0.15(5)	–
	Wt. fraction	0.416(8)	0.438(26)	0.384(16)	–
CaCO_3_ ($R\bar{3}c$)	*a* (Å)	–	–	4.961(3)	4.930(3)
	*c* (Å)	–	–	17.038(7)	16.937(6)
	Volume (Å^3^)	–	–	363.21(22)	356.50(21)
	Size (nm)	–	–	58.1(4)	36.4(15)
	Microstrain (%)	–	–	0.09(6)	0.20(6)
	Wt. fraction	–	–	0.351(12)	1
$\chi^{2}$		3.22	3.28	1.69	1.47
R_*p*_ (%)		4.43	4.15	3.29	3.05
R_*w*_ (%)		6.16	5.97	4.27	3.94
$R_{exp}$ (%)		3.44	3.30	3.29	3.25

**Table 2:** Fe–O bond lengths(Å) in the two crystallographically distinct FeO_6_ octahedra of CaFe_2_O_4_ derived from Rietveld refinement. Repeated values arise from symmetry-equivalent oxygen positions in the orthorhombic *Pnma* structure.

750 °C		850 °C	
Fe(1)O_6_	Fe(2)O_6_	Fe(1)O_6_	Fe(2)O_6_
2.02(1)	1.99(1)	2.00(1)	2.00(1)
2.02(1)	1.99(1)	2.02(1)	2.00(1)
2.02(1)	2.03(1)	2.02(1)	2.02(1)
2.04(1)	2.04(1)	2.02(1)	2.03(1)
1.98(1)	2.00(1)	1.99(1)	2.00(1)
1.98(1)	2.00(1)	1.99(1)	2.00(1)

**Table 3:** Raman peak parameters obtained from fitting, 850 °C.

Peak	ω (cm^−1^)	Γ FWHM (cm^−1^)	τ (ps)	*A* (a.u.)	ω/Γ
1	92.50	17.08	0.311	4.976	5.41
2	246.25	22.28	0.238	15.100	11.06
3	373.42	36.47	0.146	12.888	10.24
4	412.86	23.65	0.224	5.521	17.46
5	545.73	42.88	0.124	2.409	12.73
6	699.68	35.55	0.149	22.694	19.69
7	749.24	26.36	0.201	3.163	28.43

**Table 4:** Global Raman order parameter $Q_{\mathrm{Raman}}$ evaluated at different calcination temperatures.

Temperature (°C)	Number of Peaks (*N*)	$Q_{\mathrm{Raman}}$
550	3	∼4.30
650	7	∼5.88
750	8	∼7.42
850	7	∼15.00

**Table 5:** Textural parameters of CaFe_2_O_4_ calcined at 750 °C obtained from N_2_ physisorption at 77 K.

Parameter	Value
$S_{\mathrm{BET}}$ (m^2^ g^−1^)	5.575 ± 0.383
$S_{\mathrm{Langmuir}}$ (m^2^ g^−1^)	7.228 ± 0.328
$V_{t}$ (single point, $P/P_{0}\approx 0.98$) (cm^3^ g^−1^)	0.03080
$V_{\mathrm{BJH}}$ (adsorption branch, 1.7–300 nm) (cm^3^ g^−1^)	0.05064
$D_{\mathrm{BJH}}$ (adsorption branch, nm)	60.85

## Data Availability

The raw data supporting the conclusions of this article will be made available by the authors on request.
